# Editorial: Integrating Visual System Mechanisms, Computational Models and Algorithms/Technologies

**DOI:** 10.3389/fbioe.2019.00483

**Published:** 2020-01-22

**Authors:** Hedva Spitzer, Xavier Otazu, Hagit Hel-Or

**Affiliations:** ^1^School of Electrical Engineering, Tel Aviv University, Tel Aviv, Israel; ^2^Computer Science Department, Computer Vision Center, Autonomous University of Barcelona, Barcelona, Spain; ^3^Department of Computer Science, University of Haifa, Haifa, Israel

**Keywords:** computational models, algorithms, technologies, visual system, mechanisms

The Research Topic on “Integrating Visual System Mechanisms, Computational Models and Algorithms/Technologies” collects novel studies that display a strong synergy between three entities: (1) the visual system from its various angles including physiological, psychophysical, and perceptual, (2) computational models whether descriptive or predictive, and (3) vision inspired algorithms and applications. The interaction between modeling and the various aspects of the visual system is expressed in the reciprocal contributions between the two. On one hand, visual mechanisms and neuronal units provide inspiration and basis for modeling approaches and their computational units within, and on the other hand, modeling provides novel insights and new understandings of the visual system mechanisms and its associated behaviors. Furthermore, computational models, and the underlying visual mechanisms, provide a basis for developing practical algorithms to perform image processing and image understanding.

The articles in this Research Topic present computational models of the visual system ranging from neuronal mechanisms, through visual mechanisms, to visual perceptual behavior and visual illusions. Modeling efforts take different computational approaches from building blocks that are inspired by mechanisms of the visual system, to a more global Gestalt approach that attempts to explain a phenomenon regardless of the underlying elements using functional, statistical, or learning approaches. Other articles develop applications ranging from visual system inspired measures such as image quality and image esthetics to applications such as classification and segmentation.

Several studies in this issue, present computational models of the visual system at the neuronal level, and some include feasible physiological components in the model. In Gonzalez and Tsotsos, the authors suggest a computational model of attention based on the adaptation mechanisms and selective tuning of the V4 neurons which is expressed in the neurons' firing rate during attentional tasks. Different computational models are tested, coinciding with different interpretations of the attention mechanism: (a) enhancing responses due to attention or (b) suppressing irrelevant signals. The authors follow a model of the second type and are able to predict the temporal profiles of neurons' firing rate, similar to those found electrophysiologically. Through their modeling, the authors show that high level vision processes can also be explained by low-level processes, namely, that selectively tuning a model of attention, can reprsoduce properties of neuron firing rates related to attention. In another article Banerjee et al., the authors propose a computational model, based on the extreme value theory, for the integration of two sensory modalities, namely, the olfactory input and visual sensitivity of zebrafish. The authors show that the neural signals (pattern and rate of neuronal firing) differ in their statistical fit when the signals are uni-modal (visual) or multi-modal (visual + olfaction). They further showed this by developing a Machine Learning based classifier that was able to successfully distinguish between these neural signals. This study forms a contribution to the intriguing area of interactions between different sensory modalities.

Two additional articles deal with the chromatic properties of the visual system as expressed in the retinal layer and cortical layers. In Barkan and Spitzer, a computational model is presented which suggests an explanation of the underlying visual mechanisms for compensating chromatic aberrations. The computational model takes into account the spatio-chromatic properties of the color-coded cells in the retina while taking into account the significance of the anatomical separation of the Konio and Parvo chromatic pathways in the visual system. Furthermore, the model predicts the enigmatic phenomenon of S-cone pattern reported by Shevell and Monnier. In a review article, by Patterson et al., the authors discuss the role of retinal midget RGC cells and cortical double opponent cells in the context of hue perception on one hand and spatial perception on the other. The authors present hypotheses that in some form are not in accord with those supported by some other models including that of Barkan and Spitzer mentioned above. As usual in Science, especially in neuroscience, conflicting results are always an interesting source for promotion of discussion and comparison of opposite/different ideas.

Another group of studies develop computational models in order to assist in understanding specific vision mechanisms. In Piu et al., the authors acquired experimental data and then performed statistical analysis on the data to obtain a representation of pupil size changes. They analyzed oscillatory dynamics of the pupil at rest by extracting features from the cross-recurrences of these oscillators as expressed in the power spectrum. The authors state that their novel analysis approach can form an adaptable diagnostic tool for identifying alertness and/or pathological status and thus might assist in clinical assessments of pathologies associated with the autonomous nervous system. In Reynaud and Hess, the authors analyze their previously measured dataset and assess the visual disparity sensitivity of subjects across different spatial frequencies. The computational factor in their study is the data analysis methods in which they applied inter-correlations and factor analysis on the data and found two spatial frequency channels for disparity sensitivity: one tuned to high spatial frequencies and one tuned to low spatial frequencies. The authors suggest that this tuning of disparity channels could be important in computer vision to design multi-scale stereo matching algorithms. In Marić and Domijan, binary attention maps are modeled using a recurrent competitive network with excitatory-inhibitory nodes. The model reproduces top-down mechanisms of attentions that enhance perceived saliency of low-level features. The model is based on an extension of previously suggested Winner Take All (WTA) choice models, and is inspired by neurological components such as dendritic non-linearity that act on the excitatory units and modulate synaptic transmission. The model integrates a large set of data in visual attention and successfully predicts several attentional effects including the ability to integrate information across space and time to form the intersection or union of two maps that are defined by different features.

Finally, a selection of articles uses computational models to predict and explain high level visual tasks, perceptual behavior, and visual phenomena. Some of these studies experiment with ambiguous stimuli and suggest explanations of visual system mechanisms that contribute to the stabilization of the visually perceived display content. The article Cohen-Duwek and Spitzer, models the Filling-In phenomenon and, specifically, the alternating effects in which the background of a stimulus may lead to two different types of perceived color: original or complementary color. The model successfully predicts both effects through a heat diffusion function that is triggered by both the chromatic edges of the stimulus and the achromatic *remaining* contours, in contrast to previous studies that use the edges as blockers for diffusion and not as triggers. In another article Cohen-Duwek and Spitzer, a computational model is presented that predicts spatial Filling-In effects such as the Watercolor illusion and the Cornsweet effects, that have several chromatic edges. The model is based on the heat diffusion equation where the scene gradients serve as heat sources. The model successfully predicts both the assimilative and non-assimilative watercolor effects, as well as additional Filling-In visual effects. The study thus supports the theory that a shared visual mechanism is responsible (or partly responsible) for the vast variety of the “conflicting” filling-in phenomena. Two articles studied motion integration using bi-stable moving visual stimuli that can induce two different percepts (e.g., coherent and transparent). In Li et al., a bi-stable moving visual stimuli of line segments was presented to participants and their individual biases were modeled using a Bayesian modeling approach indicating a preference for one of the two possible interpretations of the scene. The authors found that increasing density shows increasing bias in observers and that this effect is greater in regular patterns than in irregular patterns. The authors tested a number of Bayesian models and show that a motion segregation prior best explains the interaction of density and regularity observed in the collected experimental data. The authors suggest that bias is used by observers to stabilize visual perception of the world. In the article Liu et al., motion integration in normal observers was compared to integration by observers with Anisometropic Amblyopia, a neurodevelopmental disorder of the visual system. They showed that when the stimuli contrast is reduced, the control observers exhibit a change in percept patterns, but amblyopic eyes do not. Using Baysian modeling, the authors show that indeed contrast affects motion integration. Considering this together with the modeling outcomes, the authors suggest that there is a different motion coding mechanism in the amblyopic visual system. Finally, in Yankelovich and Spitzer, Boundary Completion was modeled, using a functional optimization approach in which there is no need to extract different image features. The model evaluates several possible interpretations of the input and assigns a cost to each. The interpretation with minimal cost is the model's output. The model successfully predicts real and illusory contours. Additionally, for ambiguous stimulus, the model is able to find multiple possible image interpretations, which are ranked according to the probability they are perceived.

A different group of papers in this special issue, propose practical algorithms and applications that were inspired by elements of the Human Visual System, or include components that do so. In Tsitiridis et al., the authors attempt to develop a system to detect “Presentation Attacks” where a person's image is illegally reproduced and used to abuse a biometric system. The authors develop a biologically-inspired presentation attack detection model, based on features that mimic neurobiological processes in the human visual system. Machine learning tools are exploited to successfully predict whether incoming data is a spoofing-attack or is a legitimate image. In the article Paulun et al., a new system for dynamic visual recognition is introduced that combines bio-inspired sensor and hardware with a brain-like spiking neural network that mimics the layered structure and the retinotopic organization of the retina and visual cortex. Following training, the network showed a very high object classification accuracy. Finally, two papers in this group deal with image quality and esthetics. In Martinez-Garcia et al., the authors address the important question of biased or imbalanced datasets and their effect on quantitative modeling of the visual system. The authors show this in a specific case of layered retina-cortex models that learn to predict subjective quality ratings of images. They show that the database under-represents certain stimuli (such as cross-masking between different frequencies) and thus the model trained on this database does not generalize well. The authors show that by augmenting the database with synthetic examples, the model shows significant improvement in performance and generalization. The authors impress that naturalistic databases should be combined with artificial stimuli to improve model performance.

In the comprehensive review Brachmann and Redies, the authors describe the advances achieved by the Vision Science and the Computer Vision communities in the parallel fields of experimental visual aesthetics and computational visual aesthetics. The paper highlights the similarities between the types of features exploited for these tasks by both communities and the similarities between the quantitative tools used to analyze and define these features. The review covers models and algorithms that supply prediction of ratings, style, and artist identification as well as computational methods in art history of painting and photograph images. The review covers methods at both sensorial (low-level bottom-up) and cognitive levels (high-levels), including modern methods of deep learning. In addition, the review summarizes results from the field of experimental aesthetics and deal with several specific image properties. The authors show that a close interaction between computational and experimental approaches are fundamental to answering difficult questions.

In this special issue, we have collected a variety of articles that look at the intriguing cycle of: visual system, computational models, and applications. The studies show how computational models can explain the vision system from the neuronal level to the behavioral level providing understanding, and novel insights. On the other hand, the visual system provides ideas and inspiration for the computational units and driving rules of the models. The interaction cycle continues with the design of practical algorithms and applications in the field of computer vision, that arise from the computational models and the ensuing understanding of the visual system. Some of the papers in this collection, even succeeded in achieving algorithms that perform on par with state-of-the-art capabilities, due to the adoption of ideas from the visual systems. Other papers provide inspiration for future possible algorithms to accomplish different visual tasks.

Within this cycle of mutual contributions, we can learn some intriguing ideas and raise interesting questions.

A recurring notion is the idea of the visual system providing educated guesses on the visual scene, based on the visual input as well as on priors, and internal representations and computations. Multi-stable inputs in the 3D world, occluded and ambiguous scenes, allow several interpretations. However, these are processed by the visual system that considers the possible interpretations and produces an “educated guess” as the best explanation of the visual scene. Such a mechanism tends to lend stability and consistency to our visual world.

An interesting insight that has been previously established, is the importance of visual illusions as a basis for research on the visual system. As several of the articles in this issue have shown, illusions serve to mirror “errors” and “biases” of the visual system as well as provide a window into the visual system's mechanics via visual perception.

Finally, we note that several of the articles introduce the notion of aesthetics of the visual scene and raise the point that beyond a comprehensive review, a small step has been taken toward the famous philosophical-psychophysical problem also regarding to visual aesthetics through the discussion of originality and creativity.

## Author Contributions

All authors listed have made a substantial, direct and intellectual contribution to the work, and approved it for publication.

### Conflict of Interest

The authors declare that the research was conducted in the absence of any commercial or financial relationships that could be construed as a potential conflict of interest.

